# No effect of blood sampling or phytohaemagglutinin injection on postfledging survival in a wild songbird

**DOI:** 10.1002/ece3.2112

**Published:** 2016-04-03

**Authors:** Emerson Keith Bowers, Scott K. Sakaluk, Charles F. Thompson

**Affiliations:** ^1^ Behavior, Ecology, Evolution, and Systematics SectionSchool of Biological SciencesIllinois State UniversityNormalIllinois61790‐4120

**Keywords:** Capture‐mark‐recapture, house wren, recruitment, *Troglodytes aedon*

## Abstract

The injection of phytohaemagglutinin (PHA) and sampling of blood are widely used in studies of wild vertebrates to assess components of immune and endocrine function and health state and to obtain genetic material. Despite the pervasive use of these techniques in the life sciences, their potential effects on survival are rarely considered. For example, whether injection of the immunogen PHA into body parts critical for locomotion (e.g., the prepatagium, or wing web, in birds) affects survival has not been tested. Here, we test whether injection of PHA into the wing web and blood sampling from nestling house wrens affects their subsequent recruitment and survival as breeding adults. Capture‐mark‐recapture analysis on a large sample of young (*N *=* *20,152 fledglings from 3959 broods) treated over 10 years revealed that neither PHA injection nor blood sampling affected individual survival and detection probability. Recruitment as a breeder varied among years, but this variation was not attributable to sampling effort, or the percent of all adults identified at the nest during a given year. Variation in the percent of adults identified was primarily attributable to the effect of nest depredation on our ability to capture nesting pairs. Our results indicating lack of an effect of blood sampling and immune stimulation on survival are encouraging, but we recommend further work to assess the potential negative effects of all commonly used techniques on the survival of study subjects in the wild, including the potential costs associated with mounting various immunological responses.

## Introduction

The injection of phytohaemagglutinin (PHA) and blood sampling are widely used in studies of wild vertebrates. Although the use of these techniques is critical to testing various hypotheses related to the ecology, physiology, and evolution of free‐living animals, the potential effects that such techniques have on survival warrant further scrutiny. Tests of whether blood sampling affects individual survival in natural populations have been inconclusive. Traditionally, it has been commonly accepted that sampling blood using proper precautions has little or no effect on the survival of adult and developing birds (Lancot 1994; Lubjuhn et al. 1998; Schmoll et al. 2004; Sheldon et al. 2008; Angelier et al. 2011; Redmond and Murphy 2011) and small mammals (Swann et al. 1997). However, Brown and Brown (2009) reported that sampling even small amounts of blood (0.3–1.2% of body mass) from adult cliff swallows (*Petrochelidon pyrrhonota*) caused a 21–33% reduction in annual survival. At the very least, these recent findings suggest some degree of species specificity in the effects of blood sampling, obliging investigators using these techniques to determine whether they affect the survivorship of experimental subjects.

Even less is known about effects of PHA injection on survivorship in the wild. PHA is a plant‐derived lectin that induces inflammation and swelling upon injection. The injection of PHA induces a proinflammatory response, induced by cytokine signaling, and the local infiltration of leukocytes (Martin et al. 2006; Vinkler et al. 2014; Bílková et al. 2015). The magnitude of this swelling is generally accepted as a reflection of the cutaneous immune activity in adult and juvenile birds (Martin et al. 2006; Forsman et al. 2010; Vinkler et al. 2014), and the magnitude of the swelling induced by PHA among nestlings has been found to predict their recruitment as breeding adults into local populations in a number of species (Cichoń and Dubiec 2005; Moreno et al. 2005; López‐Rull et al. 2011; Bowers et al. 2014). Indeed, this test has become routinely used in a wide variety of vertebrate taxa (e.g., Martin et al. 2006; Paitz et al. 2010; Demas et al. 2011), yet the consequences of PHA injection on survival in the wild have not been studied. Injection of PHA was reported not to affect heat shock proteins that are associated with stress in nestling house martins (*Delichon urbicum*; Merino et al. 1999), but does cause temporary inflammation, swelling, and induration (Lochmiller et al. 1993). Treatment with PHA also elevates metabolic rates in birds responding to the challenge (Martin et al. 2003), potentially inducing survival costs aside from those brought about through hindered flight performance. Moreover, inflammation can often alter an individual's hematological state and other forms of physiological stress (e.g., Buchanan et al. 2003; Adelman et al. 2010; Vinkler et al. 2010; Bílková et al. 2015; Bowers et al. 2015). Although these effects may be short‐lived, their collective potential to hinder flight performance and, ultimately, survival in the wild has not been fully investigated.

**Figure 1:**
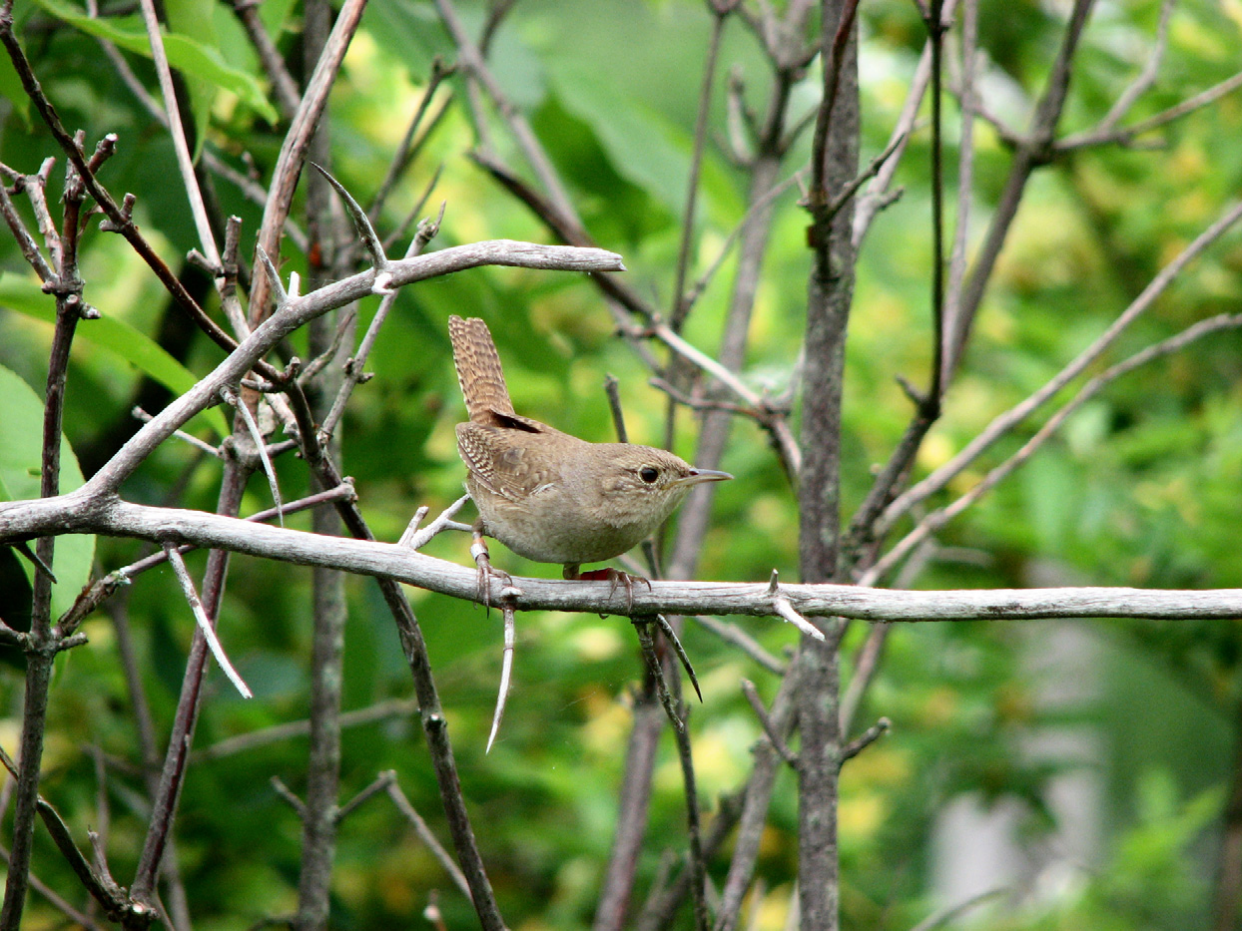
A house wren. Photograph credit: Paulo E. Llambías.

Here, we investigate whether injection of PHA into the wing web and sampling blood from the brachial vein in nestling house wrens (*Troglodytes aedon*; Fig. 1) affects long‐term survival in a local population. We also assess interannual variation in offspring recruitment rates, and how this variation is affected by sampling effort and the probability of detection. We show that variation in the probability of detecting recruits does not vary with their prefledging treatment (i.e., PHA injection or blood sampling) and that these treatments have no effect on their subsequent recruitment and survival.

## Materials and Methods

### Study area and species

House wrens are small, secondary‐cavity‐nesting songbirds with a widespread distribution in North America (biology summarized by Johnson 2014). We studied a population breeding in Illinois, USA (40.665°N, 88.89°W), that has been under continuous study since 1980. Birds in the study population winter in the southern United States and northern Mexico. The sexes do not show appreciable differences in return rate as recruits (Bowers et al. 2014), or in their natal dispersal distances in the population (median distances: females = 674 m, males = 608 m; Drilling and Thompson 1988). House wrens readily accept nestboxes for nesting, and the boxes in this study (*N *=* *820) were spaced 30 m apart along north–south transects separated by 60 m, and mounted atop 1.5‐m poles. At the start of this study (2004), all poles had been coated with axle grease to discourage terrestrial predators, but, over the next 6 years, 48.3‐cm‐diameter predator baffles were gradually added until all boxes were mounted atop baffles by the end of the 2010 field season (further details on nestboxes in Lambrechts et al. 2010). Adults in the study population also strongly prefer nesting in boxes over natural tree cavities, as ca. 95% of nests in any given year are produced in the boxes (Drilling and Thompson 1988).

### General procedures

Adults were captured from mid‐May through mid‐August during the 2004–2015 breeding seasons using a permanently mounted trapdoor on the nestbox or a mist net placed briefly in front of the entrance. We waited to catch adults until at least halfway through the incubation period to reduce the possibility of nest abandonment in response to capture and handling; thus, most adults at nests that failed prior to that time were not identified and attributed to these nests.

**Figure 2:**
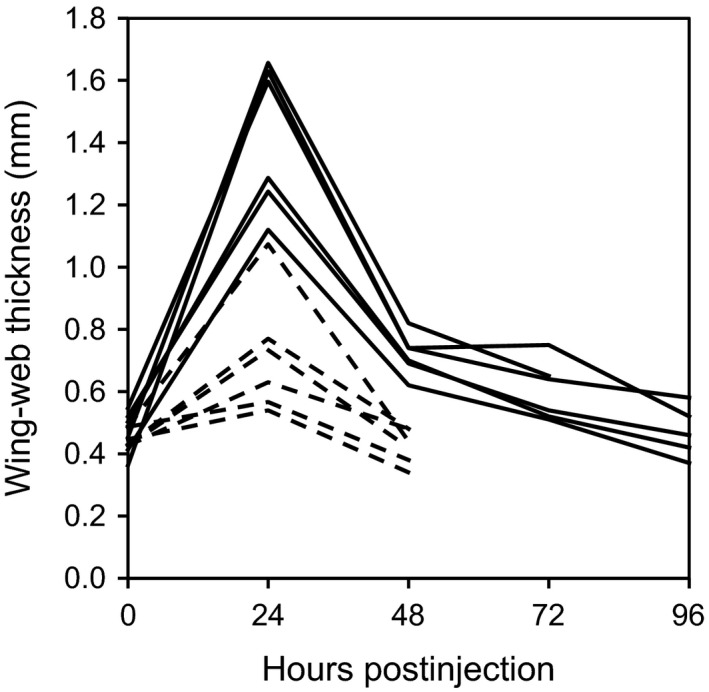
Individual variation in wing‐web thickness prior to injection with phytohaemagglutinin (0 h postinjection) and on subsequent days following injection. Plotted are individual reaction norms for 12 nestlings from two broods (solid and dashed lines represent nestlings in different broods).

The nestlings in this study (Table 1) were produced from mid‐ to late‐May through August and early September during the 2004–2013 breeding seasons (sampled and nonsampled nestlings were represented across the full, natural duration of the breeding seasons). Eleven days after hatching began within a nest (brood‐day 11), all nestlings were weighed and banded. Most nestlings were neither bled nor injected at this time (Table 1). Other subsets of nestlings were either injected with PHA, bled, or both injected with PHA and bled (Table 1). For the PHA test, we measured the thickness (±0.01 mm; Mitutoyo no. 547–500; Mitutoyo, Aurora, IL) of the wing web as the mean of three measures prior to and 24 h after the injection of the web with a 50‐μL solution of PHA (L8754; Sigma‐Aldrich, St. Louis, MO) dissolved in phosphate‐buffered saline (PBS; concentration = 5 mg PHA/mL PBS). The magnitude of nestlings' responsiveness to PHA positively predicts their recruitment as breeders in the study population (Bowers et al. 2014; for other species see Cichoń and Dubiec 2005; Moreno et al. 2005; López‐Rull et al. 2011). When blood sampling, we swabbed the nestlings' wing with ethanol prior to puncturing the brachial vein using a sterile lancet and collecting blood samples in heparinized capillary tubes. Bleeding generally stopped naturally after collection of a small blood sample (generally 10–30 μL). In rare instances, bleeding did not stop naturally, and styptic powder was applied and prevented further loss. Although we did not measure the volume of blood lost by the birds we sampled, samples were typically less than 50 μL. Processing a brood of nestlings usually took between 10 and 30 min. After processing nestlings, we returned to nests daily as fledging approached, which typically occurs on brood‐day 14–17, to monitor fledging success. We generally did not handle nestlings after measuring their responses to PHA on brood‐day 12. However, in 2004, we visited two nests, each with a modal brood size of six nestlings, for multiple days following the PHA injection to document changes in wing‐web swelling. Nestlings injected with PHA exhibited pronounced variation in their responses to the lectin, even in the small sample (Fig. 2). Although all nestlings exhibited swelling of the injected wing web after 24 h, this generally returned to pre‐injection levels by 48‐h postinjection (Fig. 2).

**Table 1:** Sample of nestlings that were injected with phytohaemagglutinin (PHA), bled, both injected and bled, or neither bled nor injected from 2004 to 2013

	2004	2005	2006	2007	2008	2009	2010	2011	2012	2013	Total
Injected only	0	0	265	0	17	0	0	0	0	0	282
Bled only	14	249	0	0	14	215	651	0	205	139	1487
Injected and bled	435	771	954	316	424	304	110	1135	912	1084	6445
Neither bled nor injected	1701	695	796	1997	2095	2098	1310	631	338	277	11,938
Total	2150	1715	2015	2313	2550	2617	2071	1766	1455	1500	20,152

### Data analysis

We attempted to identify all adults in each year preceding, during, and following the study, and to capture and band nearly every individual in the population in each year. We did not obtain data on dispersers breeding outside of the study population, and it is possible that PHA injection or blood sampling may affect site fidelity. Of 778 fledglings recruiting to breed in the population, 128 of them (16.5%) had a “gap year” (i.e., they were banded as a nestling in year *t*, and captured in year *t*_+2_ or later, but were not captured during year *t*_+1_). We previously found that whether this occurs is unrelated to nestling condition (size‐adjusted body mass) and health state prior to fledging (Bowers et al. 2014). Here, we assessed whether bleeding and PHA injection affected the probability that a recruited bird would have a gap year using generalized linear models in SAS 9.3 (SAS Institute Inc., Cary, NC) with a binary response and logit link, similar to a logistic regression. We assessed the probability of a recruit going undetected in relation to annual variation in adult capture rates. In most years, we identified a high percentage of adults at successful nests (Table 2), although the percentage of adults captured at all nests was generally lower, as many nests failed before we had an opportunity to identify the resident pair (Table 2). Thus, we analyzed the probability of a recruit going undetected as an adult for at least 1 year in relation to the percent of adults that were captured at all nests in the population using a generalized linear mixed model (GLMM) with a binary response and logit link, with year as a random effect. We also determined whether success in identifying the adults at all nests varied with rates of nest failure. Then, we analyzed whether a recruit would have a gap year in relation to its bleeding and PHA injection treatment using a generalized linear model (as above); models analyzing this as a GLMM with year and nest of rearing would not converge. Thus, we assumed recruits to represent independent observations.

**Table 2:** Adults captured in years following injection and blood sampling of nestlings

	2005	2006	2007	2008	2009	2010	2011	2012	2013	2014
Number of adults captured at all nests (% of possible captures)^a^	738 (51.5)	840 (47.6)	908 (66.7)	954 (55.2)	1022 (62.9)	958 (69.4)	806 (67.2)	800 (75.8)	883 (74.0)	806 (84.5)
Number of adults captured at successful nests (% of possible captures)^b^	555 (79.7)	631 (78.3)	713 (82.1)	714 (69.7)	799 (77.7)	728 (86.1)	569 (82.5)	500 (93.3)	540 (93.8)	633 (95.9)
Number of successful nests (% of total nests)	348 (48.6)	404 (45.8)	445 (65.3)	512 (59.3)	514 (63.3)	423 (61.3)	345 (57.5)	268 (50.8)	288 (48.2)	330 (69.2)
Total number of nests initiated	716	883	681	864	812	690	600	528	597	477

Percent of possible adult captures assumes two resident birds at each nest, which is not always the case.

a Includes clutches initiated and then failed prior to having an opportunity to capture the resident pair.

b Nests that fledged at least one young.

To analyze interannual survival, we used capture‐mark‐recapture (CMR) models in MARK 8.0 following White and Burnham (1999) and Angelier et al. (2011) to analyze comprehensive capture histories across years for all individuals in the study. These models included two main parameters: the probability of survival (Φ) and of being resighted (*P*). Thus, the probability that an individual died or emigrated is (1 − Φ), and the probability that an adult bred either in the study population or at another site, but was not identified by us, is represented by (1–*P*). We generated nine total CMR models that differed in their effects on survival and resighting (Table 3), and used sample‐size‐adjusted Akaike's Information Criterion (AICc), as provided in MARK, to determine which models were most parsimonious. We obtained differences in AICc (ΔAICc) between models, which generally determine the models providing the most acceptable description of the data.

**Table 3:** Model selection using Akaike's Information Criterion (AICc) to determine the influence of bleeding and phytohaemagglutinin (PHA) injection (i.e., treatment) on the survival and recapture probability of house wren fledglings

Model no.	Model	AICc	ΔAICc	*w*	*k*	Deviance
1	Φ(.)*P*(.)	9482.36	0.00	1.0000	65	1047.71
2	Φ(*t*)*P*(*t*)	9514.17	31.82	0.0000	21	1167.89
3	Φ(treatment)*P*(*t*)	9563.58	81.23	0.0000	15	1229.32
4	Φ(.)*P*(*t*)	9580.60	98.24	0.0000	12	1252.35
5	Φ(*t*)*P*(treatment)	9592.64	110.29	0.0000	15	1258.38
6	Φ(*t*)*P*(.)	9603.90	121.54	0.0000	12	1275.64
7	Φ(.)*P*(treatment)	9663.40	181.04	0.0000	5	1349.16
8	Φ(treatment)*P*(.)	9663.44	181.08	0.0000	5	1349.20
9	Φ(treatment)*P*(treatment)	9666.06	183.70	0.0000	8	1345.81

Φ and *P* denote the probability of survival and resighting, respectively. The term (.) indicates no effect on survival or resighting probability, whereas (treatment) indicates an effect of blood sampling or PHA injection, and (*t*) indicates an effect of year. *w* is the AICc weight, reflecting the probability of a given model being the best among those considered; *k* is the number of parameters.

## Results

**Figure 3:**
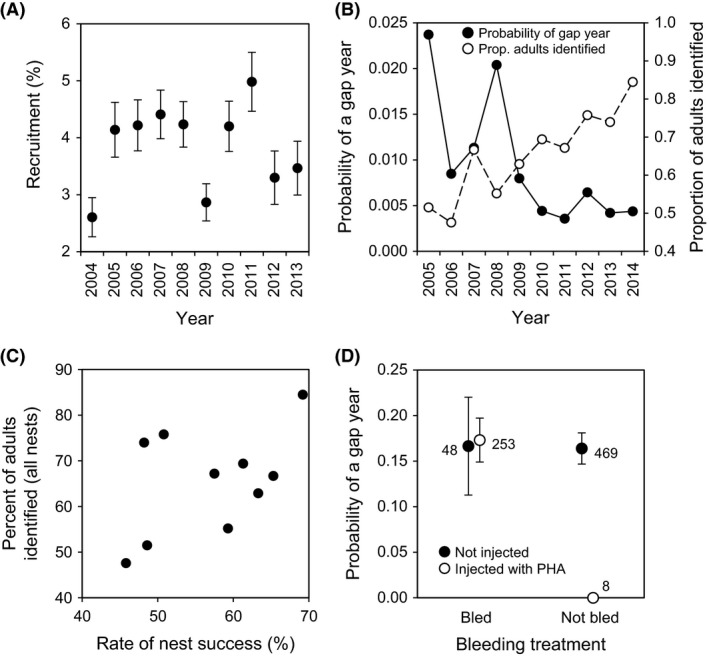
(A) Variation in the recruitment of young as breeding adults in relation to the year in which they were produced. (B) Annual variation in the probability of having a gap year (i.e., being captured for the first time as an adult at 2 years of age or older; filled symbols and left vertical axis) and the proportion of adults identified at all nests in the population (open symbols and right vertical axis). (C) Percent of adults captured at all nests in relation to annual variation in nest success (percent of nests that fledged at least one young). (D) Probability of a gap year in relation to treatment prior to fledging (least‐squares means ± SE; numbers next to points reflect the number of recruits from each group). Total sample sizes of nestlings in each group are reported in Table 1.

Recruitment of fledglings as breeding adults varied across years (Fig. 3A), and the percentage of breeding adults we were able to capture at the nest also varied across years, increasing over the course of the study (Table 2, Fig. 3B). This raises the possibility that the probability of detecting recruits varied across years, independent of recruitment *per se*. The probability that a recruited yearling would go undetected by us (i.e., have a gap year, see Data analysis) was negatively correlated with the percent of adults that were identified (GLMM: estimate ± SE = −3.636 ± 1.384, *F*_1, 9.99_ = 6.90, *P *=* *0.025; effect of year [random effect]: estimate ± SE = 0.101 ± 0.098, likelihood‐ratio test $\chi_{1}^{2}=3.42$, *P *=* *0.032; Fig. 3B). This interannual variation in adult capture rate was most likely caused by between‐year differences in nest depredation, as the percent of adults that were identified was positively associated with rates of nest success, although this trend was marginally nonsignificant (*r*_8_ = 0.475, *P *=* *0.165; Fig. 3C).

The probability of a recruit having a gap year did not differ between individuals on the basis of their having been bled or injected with PHA prior to fledging (bleeding main effect: estimate ± SE = 13.01 ± 514.56, *F*_1, 774_ = 0.00, *P *=* *0.980; injection main effect: estimate ± SE = 0.051 ± 0.421, *F*_1, 774_ = 0.00, *P *=* *0.980; interaction: estimate ± SE = 12.974 ± 514.56, *F*_1, 774_ = 0.00, *P *=* *0.980; Fig. 3D). Moreover, the probability of recruitment also did not vary with the percent of adults that we were able to identify in subsequent breeding seasons (estimate ± SE = 0.254 ± 0.650, *F*_1, 8.48_ = 0.15, *P *=* *0.706). Thus, variation in recruitment rates and detection probability across years could only introduce noise, not a bias, to our results for effects of bleeding and injection on survival.

**Figure 4:**
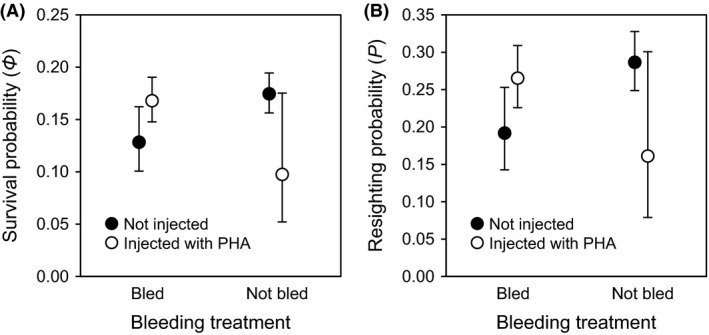
(A) Probability of survival ± 95% C.L. in relation to treatment, estimated from Model 8 (Table 3). (B) Resighting probability ± 95% C.L. in relation to treatment, estimated from Model 7 (Table 3). Total sample sizes of nestlings in each group are reported in Table 1.

The best model in describing variation in interannual survival of fledglings (Model 1, Table 3) assumed constant survival and resighting probabilities with respect to bleeding, PHA injection, and year. Despite having the highest number of parameters, this model was much more parsimonious than all others. The next best model (Model 2, Table 3), which assumed an effect of year on survival and resighting probability, but no effect of blood sampling and PHA injection, had a value of ΔAICc greater than 30, indicating that it was not nearly as close to being supported by the data. Therefore, neither blood sampling nor PHA injection affected survival (Fig. 4A) or resighting probability (Fig. 4B). Analyzing the probability of postfledgling recruitment as an adult into the breeding population as a binary outcome (recruited vs. not recruited) using a GLMM produces a qualitatively similar result (Appendix S1).

## Discussion

Approximately 24‐h postinjection, PHA induced a pronounced swelling in nestlings' wing webs, which then subsided by 48‐h postinjection (Fig. 2). Based on studies of adults and juveniles of other species, we expected that the PHA‐induced swelling in nestlings might persist into the fledgling stage, and potentially hinder the flight ability of recently fledged young. For example, in adult and juvenile house sparrows (*Passer domesticus*), PHA‐induced skin swelling persisted for at least 48‐h postinjection (Martin et al. 2003, 2006), and this was also the case for adult tree swallows (*Tachycineta bicolor*; Ardia 2007). In contrast, the nestlings in our study population were treated with an even higher concentration of PHA than that used in those previous studies, and our repeated measures of individuals prior to fledging revealed a pronounced swelling at 24‐h postinjection that subsided over the next 24 h, so that it was at or near pre‐injection levels for most birds 48‐h postinjection. House wren young typically do not fledge until 15 days posthatching, on average (Bowers et al. 2013). Thus, it is unlikely that fledglings were hindered by swollen wing tissue upon fledging and as they learned to fly, forage for themselves, and avoid predators. Notwithstanding the reduction in swelling prior to fledging, inflammation can also alter indices of physiological stress and metabolic rates (Buchanan et al. 2003; Martin et al. 2003; Adelman et al. 2010), potentially affecting survival aside from possible short‐term effects on flight performance. If individuals in the current study incurred costs associated with physiological stress induced by the response to PHA, then we would have expected to detect such an effect in our analysis of survival. Nonetheless, we encourage further studies that assess long‐term effects of immune activation, particularly those utilizing techniques activating various components of the immune system, to assess other potential costs of these physiological processes.

We did not detect evidence of an effect of blood sampling on postfledging survival, consistent with other studies that reported no effect of blood sampling on interannual survival in adults (e.g., Angelier et al. 2011; Redmond and Murphy 2011; but see Brown and Brown 2009). However, few studies have addressed the potential effects of blood sampling on young birds at a time when they are developing flight muscles and preparing to fledge. Sheldon et al. (2008) detected a transient, site‐specific effect of blood sampling on growth and body condition (body mass adjusted for size) in hatchling European starlings (*Sturnus vulgaris*). Sampling blood from the jugular vein of young hatchlings had a detectable effect on their body condition approximately 1 week later (ca. one‐third of the way through the nestling period), but there was no effect when sampled from the brachial vein at this age, and the effect of sampling from the jugular vein on body condition disappeared later in development (Sheldon et al. 2008). There were also no effects of blood sampling, from either the jugular or brachial veins, on nestling condition when sampled at 8‐day posthatching or later in development (Sheldon et al. 2008). Given that body condition prior to fledging is often a strong predictor of survival and recruitment as a breeder in birds (Tinbergen and Boerlijst 1990; Young 1996; Both et al. 1999; Naef‐Daenzer et al. 2001; Bowers et al. 2014; Iserbyt et al. 2015), lack of an effect on postfledging survival may not be unexpected. It is important to note, however, that lack of an effect of blood sampling on prefledging body condition does not preclude an effect on postfledging survival, so further work investigating such effects in young animals is warranted. Indeed, paired effects of blood sampling and ectoparasitism (e.g., Brown and Brown 2009) may be particularly influential for altricial young that are bound to a nest and, therefore, often exposed to blood‐feeding ectoparasites for extended periods of time. Moreover, although we did not record the volume of blood collected from birds in the current study, the volume of blood lost may be an important predictor of survival in the wild (Brown and Brown 2009; but see Redmond and Murphy 2011), and we encourage researchers to record this information in future studies.

Not unexpectedly, the percentage of adults captured at nests varied from year to year, and this variation had an effect on the probability of detecting recruited adults at 1 year of age, assuming they were present in the breeding population. However, the probability of detecting a recruited adult did not vary with their treatment (i.e., blood sampling or PHA injection) prior to fledging. Among‐year variation in sampling effort, or our ability to identify adults at all nests in the study population, could therefore introduce only a modest amount of noise to our results for recruitment and survival, because the chances of a recruit having a gap year before being identified varied from just 0.35% to 2.4% among years (Fig. 3B).

In conclusion, we found no effect of blood sampling and PHA injection on the recruitment and survival of fledgling house wrens, results consistent with general patterns reported for other species. Although these results are encouraging, we recommend further work to assess the potential negative effects of sampling techniques on the survival of study subjects in the wild. Indeed, there is much to be learned from comparing and contrasting the use of various ecoimmunological techniques, including the injection of specific immunogens, with respect to their effect on individual survival. Such work would shed further light, not only on the ethical implications associated with such research methodologies, but also on the costs associated with triggering different immunological responses both within and among species.

## Conflict of Interest

None declared.

## Supporting information


**Appendix S1.** There was no effect of PHA injection and blood sampling prior to fledging on subsequent recruitment as breeding adults in the local population.
