# Supplementary material for: No effect of blood sampling or phytohaemagglutinin injection on postfledging survival in a wild songbird
Source: Ecol Evol. 2016 Apr 3;6(10):3107–14. doi: 10.1002/ece3.2112 (PMC4823147; doi:10.1002/ece3.2112)
Supplement: Supplementary file 1 — Appendix S1. There was no effect of PHA injection and blood sampling prior to fledging on subsequent recruitment as breeding adults in the local population. [file ECE3-6-3107-s001.docx]

**Appendix**

There was no effect of PHA injection and blood sampling prior to fledging on subsequent recruitment as breeding adults in the local population (Table A1; Fig. A1 below). Removal of the non-significant interaction between PHA injection and bleeding revealed similarly non-significant main effects of each treatment, and these effects were similarly non-significant with body mass excluded as a covariate (data not shown).

| **Table A1.** Effects of phytohaemagglutinin (PHA) injection and blood sampling on the recruitment of fledglings as adult breeders. | | | | | |
| --- | --- | --- | --- | --- | --- |
|  | Estimate ± S.E. | *F* | ndf | ddf | *P* |
| Injection with PHA | 0.219 ± 0.177 | 0.26 | 1 | 3,874 | 0.613 |
| Blood sampling | 0.448 ± 0.388 | 0.31 | 1 | 5,466 | 0.575 |
| Injection × bleeding | 0.655 ± 0.420 | 2.43 | 1 | 7,847 | 0.119 |
| Body mass | 0.250 ± 0.045 | 30.28 | 1 | 20,051 | < 0.001 |
| Intercept | –5.750 ± 0.468 |  |  |  |  |
| *Nest* | *0.425 ± 0.089* |  |  |  |  |
| *Year* | *0.040 ± 0.026* |  |  |  |  |
| Notes: Random effects are in italics; ndf = numerator degrees of freedom; ddf = denominator degrees of freedom. | | | | | |

**Figure A1.** Rate of recruitment of fledglings as breeders in relation to their treatment prior to fledging (least-squares means ± S.E.).
